# Nutrient Loadings to Streams of the Continental United States from Municipal and Industrial Effluent^1^

**DOI:** 10.1111/j.1752-1688.2011.00576.x

**Published:** 2011-10

**Authors:** Molly A Maupin, Tamara Ivahnenko

**Keywords:** point source, nutrients, loads, major river basins, water quality, wastewater

## Abstract

**Abstract:**

Data from the United States Environmental Protection Agency Permit Compliance System national database were used to calculate annual total nitrogen (TN) and total phosphorus (TP) loads to surface waters from municipal and industrial facilities in six major regions of the United States for 1992, 1997, and 2002. Concentration and effluent flow data were examined for approximately 118,250 facilities in 45 states and the District of Columbia. Inconsistent and incomplete discharge locations, effluent flows, and effluent nutrient concentrations limited the use of these data for calculating nutrient loads. More concentrations were reported for major facilities, those discharging more than 1 million gallons per day, than for minor facilities, and more concentrations were reported for TP than for TN. Analytical methods to check and improve the quality of the Permit Compliance System data were used. Annual loads were calculated using “typical pollutant concentrations” to supplement missing concentrations based on the type and size of facilities. Annual nutrient loads for over 26,600 facilities were calculated for at least one of the three years. Sewage systems represented 74% of all TN loads and 58% of all TP loads. This work represents an initial set of data to develop a comprehensive and consistent national database of point-source nutrient loads. These loads can be used to inform a wide range of water-quality management, watershed modeling, and research efforts at multiple scales.

## Introduction

As urban populations continue to grow and land development expands, the potential for impacts to surface-water systems increases along with the demand for water for a variety of uses including aquatic habitat, recreation, and water supply. A clear understanding of the effects of urban growth on water quality is necessary to effectively manage water resources and ensure an adequate supply to meet growing demands. Water-resource managers need reliable information about activities and practices that affect local and regional water-quality conditions, including effluent discharge into water bodies. Wastewater-effluent discharges from point sources can be rich in nutrients such as nitrogen and phosphorus, which can substantially degrade water quality.

Point sources that discharge total nitrogen (TN) and total phosphorus (TP) loads to surface-water systems are included in United States Environmental Protection Agency (USEPA) studies and are known to adversely affect water quality (USEPA, 2000, 2007), and have been found to be statistically significant predictors of stream-water quality and nutrient loads in regional and national models. Preston and Brakebill (1999) found point-source discharges of nutrients to be statistically significant (*p*< 0.005) for estimating the spatial distribution of TN loading in streams of the Chesapeake Bay watershed. In a New England watershed model (Moore *et al.*, 2004), point-source discharges from municipal and industrial wastewater treatment facilities in the United States (U.S.) and Canada were found to be an important variable in estimating TN and TP stream loads. In both of these studies, point-source discharges were identified as being an important part of the nutrient budget locally and over large areas of the investigated regions.

Defining the importance of point-source nutrient loads to surface waters is critical to local and regional water-resource planning efforts. For example, to provide the growing city of Atlanta, Georgia, with a mechanism for regional coordination of water supply, wastewater treatment, and stormwater management, a Watershed Management Plan was developed using models that integrate point and nonpoint source data for nutrients (Hummel *et al.*, 2003). The models allowed future water-quality conditions to be assessed at the 12-digit hydrologic unit code level, but considered only municipal and industrial discharges >1 million gallons per day (mgd [37.85 cubic hectometers per day]). Point-source discharges proved to be critical in estimating stream nutrient loads as part of the model calibration.

Reliable data about point-source discharges are essential to support a variety of federal, state, and local planning and assessment activities. However, these data can be quite limited in availability and quality in some areas, and compiling the data over large areas can be difficult (Zogorski *et al.*, 1990; McMahon *et al.*, 2007). Data for point-source facilities and related effluent discharges are currently available in the Permit Compliance System (PCS) (USEPA, 2006c) and Integrated Compliance Information System-National Pollutant Discharge Elimination System (ICIS-NPDES), which enables USEPA or state permitting authorities to monitor facility compliance with their permit requirements, as promulgated by the Clean Water Act of 1972 (USC, 2002). At the time this study was being conducted (2006), the ICIS-NPDES was not fully implemented and only PCS data were available. The PCS data have recognized limitations in both accuracy and consistency across states and regions due to inconsistent reporting procedures and policies, and to reported quality-assurance concerns (Zogorski *et al.*, 1990; GAO, 2000; McMahon *et al.*, 2007; USEPA, 2009). Recently, the USEPA has deployed a new utility (beta release, February 2011), called the “Discharge Monitoring Report Pollutant Loading Tool” (http://www.epa.gov/pollutantdischarges), which enables users to compute point-source loads using reported 2007 and later effluent and concentration data for permitted constituents from either PCS or ICIS-NPDES databases. The loads calculated for this study are different from loads derived from the new USEPA tool in that our loads are calculated for facilities using measured flow data but missing measured nutrient concentrations, and our loads are calculated for years prior to 2007. This study also collected additional facility flow and concentration data not found in the PCS database.

Municipal and industrial facilities are designated in the PCS database as “major” or “minor” based on a combination of criteria, including the type of facility, the magnitude of effluent discharges, the constituent pollutants and water-quality limiting factors of receiving waters, and the proximity of effluent discharges to coastal waters and downstream drinking water intakes. Classification of a facility is subject to change during each permit reissuance period, typically within five-year time frames, and the classification of a facility in this work is reflective of the PCS database at the time that data were retrieved (2006). Major facilities typically discharge on average more than 1 mgd of effluent, and minor facilities discharge on average <1 mgd of effluent. The distinctions between major and minor facilities are important in this work for assessment of facility data quality and point-source nutrient loads.

Previous efforts to compile regional (USEPA, 2006a,b; McMahon *et al.*, 2007) and national (Luken *et al.*, 1976; Gianessi and Peskin, 1984; NOAA, 1999) data on point-source loadings of contaminants have attempted, with varying levels of success, to address these data problems by applying quality-assurance checks and developing procedures for estimating, supplementing, or correcting erroneous data values. These procedures were designed to address some of the common data quality and availability issues, such as those related to the incomplete reporting of pollutant concentrations, effluent discharge, level of wastewater treatment, and discharge location, as well as the lack of information for minor facilities.

Georeferenced data for point-source discharges to rivers and streams in the U.S. are needed to support a variety of surface-water assessment activities, including Total Maximum Daily Load (TMDL) nutrient criteria evaluations, and watershed modeling of point and nonpoint pollutant sources (Smith *et al.*, 1997; Preston *et al.*, 2009). Thus, an accurate location for a facility's point of discharge on the stream network is important for appropriately relating the mass of nutrients that are discharged to downstream measurements of stream nutrient loads. In a broader context, any water-quality assessment methodology based on mass-balance principles would have similar requirements, and any large-scale assessment methodology would benefit from a consistent and spatially explicit database documenting point-source nutrient loads. PCS contains discharge locations, effluent flows, and some effluent nutrient concentrations. However, at the time this study was done there was no national database that provided point-source nutrient loads for individual facilities when reported effluent nutrient concentrations are completely absent.

**FIGURE 1:**
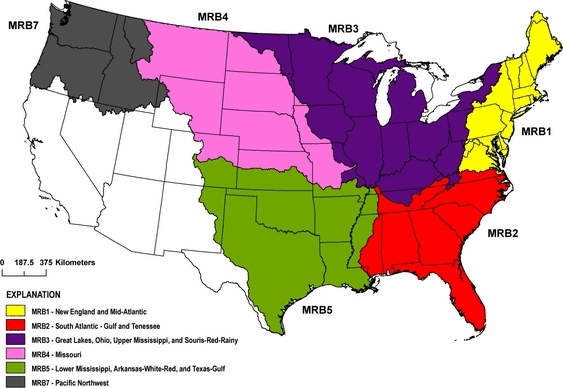
Regions (Major River Basins, or MRBs) Selected for the Development of SPARROW Nutrient Models.

To meet the need for reliable information on point-source nutrient loads to surface waters, we present a methodology for systematically evaluating and, where possible, improving the quality of the PCS data for calculating annual loadings to U.S. streams. Our approach refines the previous methods employed by the USEPA (2006a,b); and McMahon *et al.* (2007) to address specific data needs to support recent model-based assessments of nutrient sources in six major regional watersheds (called major river basins, or MRBs) (Figure 1) of the continental U.S. These regional watersheds are the focus of U.S. Geological Survey (USGS) SPARROW (SPAtially Referenced Regressions On Watershed attributes) modeling studies presented in this featured collection of the *Journal of the American Water Resources Association* (*JAWRA*) (see Preston *et al.*, this issue). The work that we describe here is also part of a broader USGS effort to develop a national database documenting point-source nutrient loads to surface waters. In this article, we describe the processes, assumptions, and enhancements to the methods initially developed by McMahon *et al.* (2007), and we summarize the data resulting from the application of those methods to the six regions. No national database currently exists for describing point-source nutrient loads using data for 1992, 1997, and 2002. In addition to supporting regional SPARROW model development, the results of this study are useful for a broad range of water-quality assessment and management activities.

## Study Methods

The primary data source for this work was the USEPA PCS database (USEPA, 2006c), a national repository of data for all municipal and industrial facilities that hold NPDES permits, which allow the release of effluent to surface-water bodies. The PCS database, the most comprehensive national point-source database available at the time of this study, is designed to compile information on permitted discharges and on the water-quality constituents included in those permits. For these reasons and for purposes of this article, we generally limit the scope of our analysis to the data available in PCS, with the exception of some additional effluent flow data and concentration obtained from several states. Because reporting requirements vary by state, region, and type of facility, the data stored in PCS in some instances were found to be incomplete, especially for minor facilities, or inaccurate with respect to effluent flows or locations. These deficiencies limited the utility of PCS data for comprehensive water-quality assessment by requiring substantial quality-assurance checks prior to load calculations. In general though, the results are limited to what could be learned by the data included in the PCS database, and in some parts of the country facilities may not be fully represented due to a virtual lack of information reported in the PCS database. Retrievals from PCS were performed by USEPA and provided to the USGS in October, 2006, and results and summaries in this article reflect the status of the PCS at that time. Facility information retrieved from the PCS database included location, size and type, effluent flows, effluent concentration, and permit information for all facilities in all states within each of the regions.

An objective of our study was to evaluate the quality of the data in the PCS database, correct data when necessary, and fill in missing data where possible to calculate annual point-source nutrient loads for each facility. PCS data for five regions were evaluated using methods that were initially developed in a pilot study (McMahon *et al.*, 2007) that estimated annual point-source nutrient loads in the South Atlantic-Gulf and Tennessee (MRB2), hereafter called the Southeast, for about 3,000 facilities and the year 2002. The pilot project approach was based on procedures used in an USEPA investigation of nutrient loading to the Mississippi River basin (USEPA, 2006a,b;). The methods and data developed in the pilot study for the Southeast (MRB2) were reported by McMahon *et al.* (2007) and by Hoos *et al.* (2008) and are summarized here. In the five other regions, annual point-source nutrient loads were calculated for facilities using reported effluent flow and effluent nutrient concentrations for the calendar years 1992, 1997, and 2002. These three years coincide with the period of collection of other nutrient data used in the regional SPARROW models (Preston *et al.*, this issue), such as county-wide fertilizer use and crop-production data from the U.S. Department of Agriculture, Census of Agriculture (USDA, 1994, 1998, 2004). The 2002 calendar year was the focus of the regional SPARROW modeling effort (Preston *et al.*, this issue), and thus summaries for point-source nutrient loads from the 2002 data alone are presented. The database we developed, however, contains facility information, effluent flow, effluent nutrient concentrations, and annual point-source nutrient loads for all three years for the five regions (excluding MRB2), and for the year 2002 for MRB2 only.

Annual nutrient loads were calculated only for those facilities with measured effluent flow, since this was considered a critical part of the load calculation. Measured effluent flow was frequently missing from the database, particularly for minor facilities, and that became a substantial limitation in the number of facilities for which point-source nutrient loads could be calculated. All facilities in each region for which effluent flow data were available were examined for quality-assurance purposes to correct errors or identify missing data. Effluent flows were analyzed for outliers, and all values that exceeded 100 mgd, or that were >10 times the median effluent flow rate, were checked, verified, and corrected if necessary. Most errors consisted of missing decimal points for records that indicated effluent flows were in millions of gallons per day, when in fact they were in gallons per day. An estimated 30% of the flow data, of which there are over half a million monthly flows used in load calculations, were either corrected or had units converted. Effluent concentration data were also checked for outliers and decimal placement errors. Additionally, facility effluent concentration data were checked for values that could be used to increase the number of TN concentration data that could be used to calculate loads. Approximately 400 facilities were identified that lacked monthly TN concentrations, but had monthly data for other constituents that could be added together to compute a monthly TN concentration value (i.e., Nitrogen, Kjeldahl total as N, and Nitrite plus Nitrate, total as N)*.* The newly computed monthly TN concentrations were calculated using the individual constituent concentrations and entered into the database to be used for point-source nutrient load estimates for those facilities.

Locations of facility discharges were also considered important since they determined the recipient streams. Locations were plotted, and the state, county, and watershed information was checked. Correcting invalid locations required research, sometimes necessitating contact with facility operators, or using state database locations obtained using Global Positioning Systems (GPS) data (William Donehoo, USEPA, Region II PCS Database Administrator, 2006, oral communication). If street addresses were known, locations were obtained by using Google Earth™ (any use of trade, product, or firm names is for descriptive purposes only and does not imply endorsement by the U.S. Government). Approximately 55% of facilities with loads had location data that was either provided through, or changed by, work in this study.

The type of facility discharging effluent is defined by a Standard Industrial Classification (SIC) code, which is a four-digit code used to classify facilities by the type of activity in which they are engaged (Office of Management and Budget, 1987). SIC codes were used to characterize facilities according to the potential magnitude of the nutrient load. A list of the SIC codes used in this study is found in Table S1 (Supporting Information), and includes codes that were used in the pilot study in addition to those from this work (McMahon *et al.*, 2007). Sewage systems are the predominant type of facility with point-source nutrient loads in this study. Additionally, steel mills and finishing plants, blast furnaces, and primary nonferrous metal facilities were determined by the USEPA to be primary contributors to nutrient loading in the Mississippi River (USEPA, 2006b), and were also included in this analysis. Some facilities were classified and assigned a code of “9999” (Nonclassifiable Establishment) in PCS. For our purposes, all facilities were re-assigned a code that most closely represented their primary activity so that they could be compiled by SIC groups without having an ambiguous set.

Facilities that lacked effluent flow, discharge location, or SIC data, especially sewage systems or other types of facilities that have the potential to contribute high nutrient loads, were prioritized for additional data requests to state regulatory agencies. The first priority was to gather any missing data for all major facilities. The collection of data for minor facilities was a secondary priority, and counties with few major facilities were given greater emphasis for investigation and collection of possible data for load calculation. The combined contribution of point-source nutrient loads from multiple minor facilities to the same surface-water body potentially represents a large source of nutrients to that water body, which would be important to document for any local water-quality assessment (USEPA, 2009).

We calculated annual point-source nutrient loads for facilities based on effluent flow and effluent nutrient concentration data using programs described in McMahon *et al.* (2007). McMahon's methods computed annual point-source nutrient loads, in kilograms per year, for facilities with effluent flow and effluent nutrient concentration data, as well as for facilities with only effluent flow. If a monthly effluent flow existed for each month of the year, loads were calculated as the flow (in mgd) multiplied by the number of days in the month and the nutrient effluent concentration (in milligrams per liter), and then converted to kilograms per month. The monthly loads were totaled over the year. If there were monthly flows for <12 months, but flows existed in three or four quarters of the year, it was assumed that the facility discharged each month of the year and seasonal median flows and seasonal median nutrient concentrations (1992, 1997, and 2002) were multiplied times the number of days in the season and totaled to an annual load. If flows were reported for less than three quarters per year, loads were calculated only for the months with effluent flow and effluent nutrient concentration data, and all of those months were totaled for an annual load. Seasons were based on winter (December-February), spring (March-May), summer (June-August), and fall (September-November).

To calculate loads from facilities that lacked effluent nutrient concentration data, McMahon *et al.* (2007) describe a hierarchical approach to develop surrogate effluent nutrient concentrations termed “typical pollutant concentrations” (TPC) based on the type (i.e., as defined by SIC) and size of the facilities. Four options are used for developing TPC values, discussed in declining order of preference:

1. If effluent nutrient concentration data were missing for one or more months for a facility, a median seasonal concentration was calculated as mentioned above using concentration data for the facility from other years (1992, 1997, and 2002), and substituted for the missing data.
2. If facility-specific median seasonal nutrient concentrations could not be derived, TPC values were calculated using nutrient concentration data from other facilities of the same size and type from within the MRB.
3. For facilities still lacking nutrient concentration data, median seasonal nutrient concentration data were calculated using facility nutrient concentration data from all MRBs, again based on size and type.
4. Finally, if none of these methods supplied an applicable TPC nutrient concentration, a national SIC-specific TPC (Steven Rubin, USEPA, 2006, written communication) value was used. These SIC-specific TPC values were generated through the USEPA's Convert computer programs and are based on a number of TPC sources including the USEPA (1988) and the National Oceanic and Atmospheric Administration (NOAA, 1999), and are nutrient concentration medians derived from 10% or greater of the facilities having PCS data available for that industry. The final set of TPC values used for this study was developed by combining reported effluent nutrient concentrations by facility type across all five MRBs (combined MRB TPC, Table 2) and estimating a median value for each.

Facilities with anomalously large or small annual point-source nutrient loads, those with inter-annual loads that exceeded the 95th percentile, or that were below the 5th percentile underwent more detailed screening to look for extreme or unrealistic nutrient concentrations or erroneous effluent flow data. In such cases, the state agency responsible for collecting PCS data was contacted for clarification, and they suggested possible corrections to the data. Not every state in the MRBs provided additional data, guidance, or review of problematic sites; fewer than 10 states provided assistance in the form of reviews or additional data. However, several instances of high point-source nutrient loads for minor sewage systems in some midwestern states were explained through discussions with state agencies and facility personnel where the local facility management practice was to hold effluent in retention ponds and release it on a quarterly basis, thereby producing high seasonal point-source nutrient loads.

## Results of Data Analysis

Facilities and their associated effluent flow and nutrient concentration data that are reported in PCS vary by state and by type of facility. In general, information for major facilities is more comprehensive than that for minor facilities because state and federal regulations commonly require more in-depth reporting for major facilities. There are more minor than major facilities in the country, but less data in PCS for the minor facilities, however, presumably because states have not transferred the reported information for these facilities from local databases to PCS, or the facilities are not required to report discharges and pollutant effluent concentrations as often, if at all. One should not assume that flow and/or concentration data are included in PCS for all facilities that discharge effluent to surface-water bodies. Based on 2007 data, PCS and ICIS-NPDES include information for approximately 86% of all major facilities with some flow or concentration data, and about 37% of all minor facilities (Carey Johnston, USEPA, 2011, written communication) (http://www.epa-echo.gov/echo/dmr_map/us/dmr_universe.html).

Data from approximately 118,250 municipal and industrial facilities in 45 states and the District of Columbia are compiled from the PCS database (Table 1). These include facilities that are designated in PCS at the time of the data retrieval (2006) as both active and inactive with some inactive facilities having effluent flows and thus point-source nutrient loads calculated for only the earlier years. Of the total facilities, only about 6% are major facilities. The Great Lakes, Ohio, Upper Mississippi, and Souris-Red-Rainey, hereafter called the Upper Mississippi (MRB3), have the largest number of facilities (65,602) and the Southeast (MRB2) has the fewest. Those differences are likely due in part to the greater size of the Upper Mississippi region, which has about 48 facilities per 1,000 square kilometers (km^2^) compared to about 10 facilities per 1,000 km^2^ in the Southeast. The density of facilities nationally is consistent with population patterns in general with the New England and Mid-Atlantic (MRB1) and Upper Mississippi (MRB3) having the largest populations and the greatest densities of facilities. Less populated regions such as the Missouri (MRB4), the Lower Mississippi, Arkansas-White-Red, and Texas-Gulf (hereafter called the Gulf Coast [MRB5]), and the Pacific Northwest (MRB7) have much lower densities. Most of the facilities, from 83 to 97% of the total number by region, were in the minor category.

**TABLE 1:** Total Numbers of Active and Inactive Facilities (October, 2006), Both Major and Minor, and Number of Facilities With Reported Effluent Flow for All Years, and 2002, by Major River Basin

		All Facilities					Major Facilities		Minor Facilities	
				
MRB	Area (km^2^)	Number of Facilities in Database (active and inactive)	Density of Facilities (#/1,000 km^2^)	Number of Major Facilities in Database	Number of Minor Facilities in Database	Percentage of Facilities That Are Minor	Number of Major Facilities With Effluent Flow for 1992, 1997, or 2002	Number of Major Facilities With Effluent Flow for 2002	Number of Minor Facilities With Effluent Flow for 1992, 1997, or 2002	Number of Minor Facilities With Effluent Flow for 2002
1	443,843	12,836	28.9	2,134	10,702	83.4	1,030	941	1,290	1,032
2	808,080	7,913	9.8	1,215	6,698	84.6	970	687	2,198	2,032
3	1,371,536	65,602	47.8	1,959	63,643	97.0	1,694	1,594	12,171	8,382
4	1,323,859	9,893	7.5	358	9,535	96.4	283	267	1,744	1,129
5	1,368,600	13,370	9.8	1,276	12,094	90.5	1,141	1,075	3,635	3,186
7	718,476	8,636	12.0	232	8,404	97.3	201	190	292	179
Total	6,050,760	118,250	19.5	7,174	111,076	93.9	5,319	4,754	21,330	15,940

Notes: Major facilities discharge on average more than 1 million gallons per day (mgd) of effluent, and minor facilities discharge <1 mgd of effluent. MRB, major river basin.

Of all the facilities listed in PCS (approximately 118,250 as of October 2006), about 22% have reported effluent flow (and therefore nutrient loads) for any of the three years. Measures of both effluent flow and effluent nutrient concentrations should be available to reliably calculate nutrient loads from a facility. TPC data can be substituted for measured effluent TN or TP concentrations based on the type of facility, if measured concentration data are not available from PCS. Effluent flow, however, cannot be reliably estimated or substituted and thus nutrient loads were not calculated for facilities lacking effluent flow data. Of the major facilities, most (44 to 84% by region) have reported effluent flow data for 2002 in PCS. Of the minor facilities, however, a much smaller percentage (2-30% by region) had reported effluent flow data for 2002 (Table 1).

**FIGURE 2:**
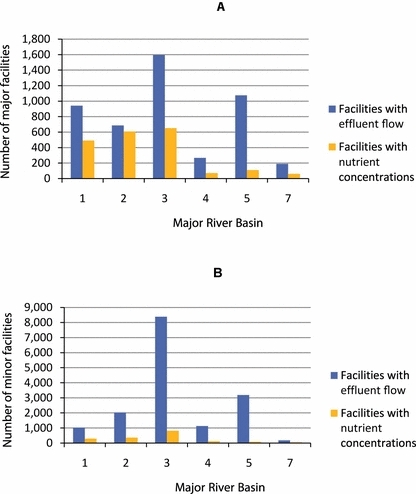
Number of Major (A) and Minor (B) Facilities With Measured Effluent Flow, and Total Nitrogen and/or Total Phosphorus Concentration Data That Were Used to Calculate Point-Source Nutrient Loads for 2002.

The percentage of facilities that reported effluent flow and effluent nutrient concentrations was highest in the Southeast (MRB2) compared to all other regions. Figure 2 illustrates by region the number of major (A) and minor (B) facilities that reported effluent flow and effluent nutrient concentrations for either TN or TP. Effluent nutrient concentrations are reported for greater percentages of major facilities than for minors, but in all regions except MRB2 the percentages of major facilities reporting effluent concentrations were <50%, and for minor facilities were <30%. More concentration values were reported for TP than for TN, because most facilities report only ammonia, and in this study, our analyses were based on TN values. These results identify a substantial data gap – the need to develop surrogate measures of nutrient concentrations in effluent for calculating point-source nutrient loads from those facilities lacking measured effluent concentration of TN or TP.

The combined MRB concentration data were compared with an independently derived set of national TPC values from the USEPA (Steven Rubin, USEPA, 2006, written communication), and in general they are in close agreement (Table 2). Where the two are the same, it is presumed that the data used to develop the USEPA TPC concentrations did not contain outliers or concentrations significantly different from those in the combined MRB data. For some facility types, however, there are large differences between the combined MRB concentration for phosphorus and the USEPA TPC concentration. Such differences may be precipitated by updates to the PCS database used to estimate the more recent combined MRB TPC values, which could have either included or omitted some extreme values. For calculating point-source nutrient loads for facilities in this study, the combined MRB concentrations were used.

**TABLE 2:** USEPA National TPC Median Values for TN and TP, and Combined MRB TPC Median Values for TN and TP That Were Used in Calculating Point-Source Nutrient Loads for Selected Types of Facilities, and Percent Differences Between the Two

		USEPA TPC^1^		Combined MRB TPC		Percent Differences	
				
SIC Code	SIC Description	TN (mg/l)	TP (mg/l)	TN (mg/l)	TP (mg/l)	TN	TP
0921	Fish hatcheries and preserves	0.7	0.09	0.7	0.05	0.000	−44.4
1221	Bituminous coal and lignite surface mining	11.2	7.0	11.2	0.3	0.000	−95.7
1311	Crude petroleum and natural gas	11.2	7.0	11.2	7.0	0.000	0.0
2011	Meat packing plants	10.8	0.91	10.8	0.91	0.000	0.0
2611	Pulp mills	1.4	0.64	1.4	0.64	0.000	0.0
2621	Paper mills	1.4	0.64	1.4	0.5	0.000	−21.9
2631	Paperboard mills	1.4	0.64	1.4	0.64	0.000	0.0
2819	Industrial inorganic chemicals, NEC	1.9	0.41	1.9	0.27	0.000	−34.1
2869	Industrial organic chemicals, NEC	3	0.34	3	0.73	0.000	114.7
3312	Steel works, blast furnaces (including coke ovens), and rolling mills	2.5	NA	2.5	0.2	0.000	NA
3334	Primary production of aluminum	8.5	NA	8.5	NA	0.000	NA
4952	Sewage systems	11.2	2.02	10.35	0.89	−7.6	−55.9

Note: TN, total nitrogen; TP, total phosphorus; TPC, typical pollutant concentrations; SIC, Standard Industrial Classification; MRB, major river basin; NEC, not elsewhere classified.

1 Steven Rubin, USEPA, 2006, written communication.

**FIGURE 3:**
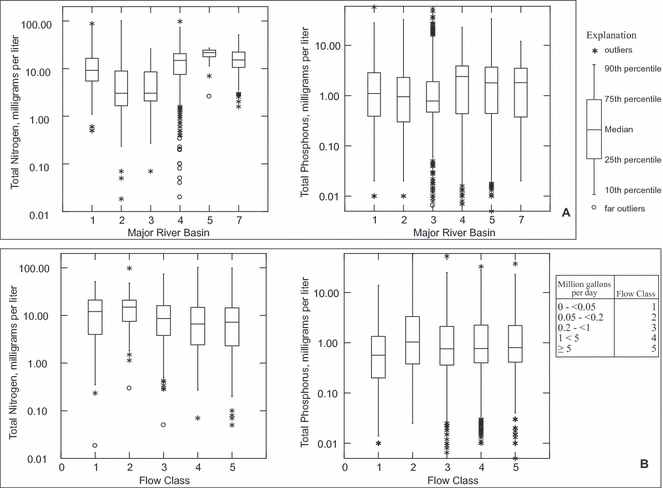
Median Concentrations of Total Nitrogen and Total Phosphorus for Sewage Systems With Reported Data in 2002 by Major River Basin (A) and Flow Class (B).

The median TN and TP concentration values for sewage systems (SIC 4952) are shown in Figure 3 by MRB and by facility size, as defined by flow class. The ranges in values that comprise 50% of the population of values are illustrated by the height of the boxes, and the median concentrations are illustrated by the central line in the boxes. Median concentrations of TN and TP are different across the MRBs, in some cases by a few milligrams per liter. The total range of concentrations between MRBs for both constituents are quite large, ranging from <0.1 to about 100 mg/l for TN, and from <0.01 to more than 10 mg/l for TP. Median TN concentrations are noticeably different between MRBs with the Southeast (MRB2) and the Upper Mississippi (MRB3) both having considerably lower median TN concentrations than the other MRBs. The Gulf Coast (MRB5) has the highest median TN concentration as well as the smallest range of values within the 50th percentile. The New England and Mid-Atlantic (MRB1) and the Upper Mississippi (MRB3) are the two most densely populated regions with some of the largest sewage systems in the U.S. However, their median TN concentrations are noticeably different from each other, most likely because our database was substantially bolstered by additional information provided by states and facilities in the Upper Mississippi (MRB3), which increased the population of data for smaller facilities there as compared to the New England and Mid-Atlantic (MRB1), which is comprised almost entirely of only major facilities.

Median TP concentrations are lowest in the Upper Mississippi (MRB3), but the Southeast (MRB2) and the New England and Mid-Atlantic (MRB1) are nearly the same. These three regions, however, all have noticeably lower median TP concentrations than the three more westerly regions; the Missouri (MRB4), the Gulf Coast (MRB5), and the Pacific Northwest (MRB7).

Median concentrations for TN and TP for different facility sizes do not appear to vary as much as they do between the different MRBs. Median TN concentrations are somewhat lower in the larger flow classes, possibly reflecting a higher level of treatment that may be required in some MRBs for larger facilities. By contrast, median TP concentrations are similar between the larger flow classes (>0.2 mgd) but have more outliers at the lower levels, possibly reducing the median. Median TP concentrations for facilities in flow class 2 (between 0.05 and 0.2 mgd) are the highest among all the flow classes. Over 80% of the facilities with TP concentrations in this flow class are in the New England and Mid-Atlantic (MRB1), the Southeast (MRB2), and the Upper Mississippi (MRB3), and have few outliers. The range of TP concentrations is much greater for the facilities in the larger flow classes (>0.2 mgd), indicating that large sewage systems across the U.S. are releasing effluent with very different levels of TP concentrations. The outliers at the lower levels for the larger flow classes are predominantly from the larger systems in the eastern regions of the U.S.

**FIGURE 4:**
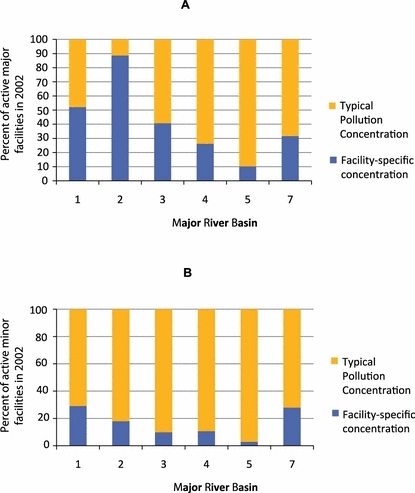
Summary of Sources of Concentration Data Used in Point-Source Nutrient Load for Major (A) and Minor (B) Facilities, by Major River Basin.

Point-source nutrient loads were calculated on a facility basis using measured effluent flow and either measured effluent nutrient concentrations or the appropriate TPC value based on the type and size of facility. TPC values were used in most cases because relatively few facilities existed in PCS with measured effluent nutrient concentration (Figure 2). Figure 4 illustrates by MRB the percentages of major (A) and minor (B) facilities for which measured nutrient concentrations or TPC values were used to calculate point-source nutrient loads. In general, more major facility than minor facility point-source nutrient loads were calculated with measured effluent nutrient concentrations, and MRB2 exhibited an especially high percentage (88). Point-source nutrient loads from minor facilities were calculated with measured nutrient concentration for 30% or fewer facilities in each MRB.

Point-source nutrient loads were calculated for over 26,600 facilities (about 22% of the total number of those listed in PCS) for at least one of the three years of interest (1992, 1997, and 2002), and for 20,694 facilities for 2002 (Table 1). The largest number of facilities (13,865) for which point-source nutrient loads were calculated is for the Upper Mississippi (MRB3). Annual point-source nutrient loads for 2002 were calculated for 17.5% of all facilities (66% of major and 14% of minor). Point-source nutrient loads were calculated for a smaller percentage of minor facilities, but the number of point-source nutrient loads for minor facilities is larger than for major facilities because there are more minor facilities in the PCS database and in the country.

Point-source nutrient loads indicate that nutrient inputs from point sources are highest where major industrial and municipal facilities are most dominant and in MRBs with large urban populations with large sewage systems (Table 3). On a regional basis, the largest amount of nutrient loading from point sources is in the Upper Mississippi (MRB3), where the amount of TN loads are more than twice that of any other MRB. However, much of that difference is due to the size of that region compared to the others. Dividing the total regional point-source nutrient loads by the area of the region provides a weighted estimate of the point-source loads, and normalizes the regions by size. On the basis of these normalized values, point-source nutrient loads (both TN and TP), on an area-weighted basis, are greatest in the New England and Mid-Atlantic (MRB1), followed by the Upper Mississippi (MRB3) and the Southeast (MRB2). The more westerly regions (Missouri [MRB4] and Pacific Northwest [MRB7]) tend to have lower point-source nutrient loads on both a total mass and on an area-weighted basis.

**TABLE 3:** Point-Source Nutrient Loads (2002) for All Facilities, and Distribution of Total Nitrogen (A) and Total Phosphorus (B) Loads Among Major River Basins

(A) Total Nitrogen (TN)						

MRB	Total TN Load (kg/year), All Dischargers	Total TN Areal Load (kg/km^2^/year), All Dischargers	Median Facility TN Load (kg/year)	Percentage of Total TN Load From Minor Facilities	Percentage of Facilities That Are Sewage Systems (SIC 4952)	Percentage of TN Loads That Were From Sewage Systems
1	120,799,415	272.2	4,985	6.5	70.7	86.0
2	56,211,267	69.6	1,450	8.0	61.2	77.6
3	252,055,066	183.7	668	13.3	50.5	74.6
4	36,888,359	27.9	906	47.4	63.6	50.7
5	87,153,571	62.9	1,470	11.5	64.6	62.6
7	23,074,312	32.1	9,992	5.7	59.1	82.4
Total	576,181,990	95.3	1,291	13.0	57.7	74.2

(B) Total Phosphorus (TP)						

MRB	Total TP Load (kg/year), All Dischargers	Total TP Areal Load (kg/km^2^/year), All Dischargers	Median Facility TP Load (kg/year)	Percentage of Total TP Load From Minor Facilities	Percentage of Facilities That Are Sewage Systems (SIC 4952)	Percentage of TP Loads That Were From Sewage Systems
1	12,845,111	28.9	386	2.4	70.7	85.3
2	8,798,645	10.8	240	12.0	61.2	66.4
3	18,920,674	13.8	109	28.0	50.5	63.6
4	7,915,609	6.0	113	59.6	63.6	27.4
5	11,833,615	8.5	190	22.4	64.6	37.9
7	4,196,267	5.8	1,665	14.3	59.1	56.0
Total	64,509,921	10.6	161	22.7	57.7	58.7

Note: MRB, major river basin (see Figure 1); SIC, Standard Industrial Classification.

Minor facilities in most regions contributed a smaller, although appreciable, part of the total regional point-source nutrient loads. Table 3 lists the percentage of the point-source nutrient loads contributed by minor facilities. Those percentages range from 5.7 to 47.4% of the total TN point-source nutrient loads generated within the regions and from 2.4 to 59.6% of the total TP point-source nutrient loads. Minor facilities had the most complete data in the Southeast (MRB2 pilot study) and the Upper Mississippi (MRB3) because of the additional effort to gather facility information from the states in those regions, but represented the greatest percentage of total loads for both TN and TP in the Missouri (MRB4). By contrast, data from minor facilities were not as complete in the Pacific Northwest (MRB7) and New England and Mid-Atlantic (MRB1). In most regions, however, minor facilities are estimated to contribute from about 6 to almost 50% of the total point-source nutrient loads, although those estimates could be even higher given that minor facilities are not fully accounted for in PCS. The large number of minor facilities and their combined potentially large contributions to the total point-source nutrient load have important implications for regional water-quality management.

**FIGURE 5:**
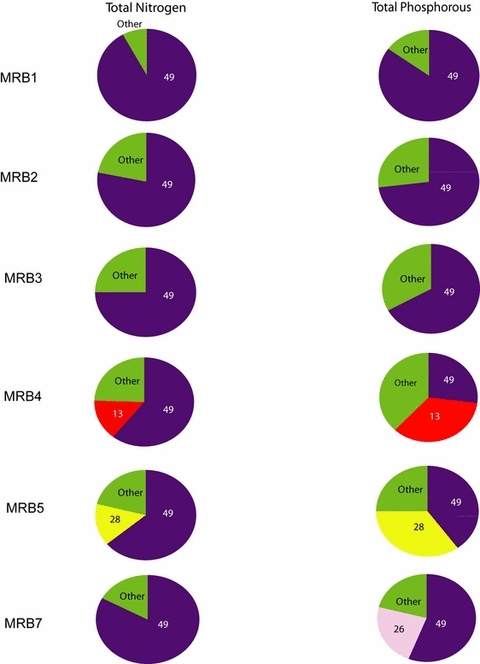
Percentage of Annual Total Nitrogen and Phosphorus Point-Source Nutrient Loads for 2002 by Major River Basin (MRB), and Standard Industrial Classification (SIC) Code. Total annual nitrogen and phosphorus point-source nutrient loads, by major river basin are shown in Table 3.

Of all the facilities evaluated as part of this study, sewage systems make up the largest numbers and contributed the largest percentage of the total point-source nutrient loads (Table 3). In all regions, sewage systems make up from 50 to 70% of the facilities for which point-source nutrient loads were calculated, and represent almost 74% of TN and 59% of TP point-source nutrient loads for all regions. Sewage systems make up the largest percentage of TN point-source nutrient loads in all regions, and the majority of the TP point-source nutrient loads generated in the New England and Mid-Atlantic (MRB1), Southeast (MRB2), Upper Mississippi (MRB3), and Pacific Northwest (MRB7) (Figure 5). These results emphasize the importance of sewage systems as a source of nutrients to surface waters and their importance as a component of any water-quality management plan.

Other types of facilities (besides sewage systems, SIC 49), however, also contribute substantially to the total point-source nutrient loads of each region (Figure 5). In the Missouri (MRB4), TP point-source loads contributed by crude petroleum and natural gas facilities (SIC 13) exceeded those of sewage systems. TP point-source loads from industrial organic and industrial inorganic chemical facilities (SIC 28) exceeded sewage system TP point-source loads in the Gulf Coast (MRB5), and TP point-source loads from pulp and paper mills (SIC 26) in the Pacific Northwest (MRB7) were a substantial portion of the total in that region. The USEPA's (2006a,b,c); point-source assessment of the Mississippi River Basin found that pulp mills, paper mills, and industrial organic and inorganic chemical facilities were the primary nonsewage facilities contributing substantially to nutrient loads in receiving waters (USEPA, 2006b).

**FIGURE 6:**
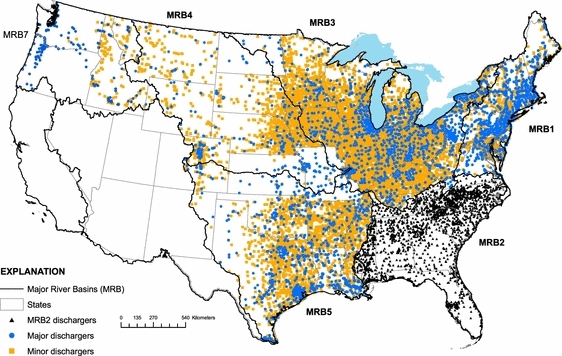
Facilities in the New England and Mid-Atlantic (MRB1), Upper Mississippi (MRB3), Missouri (MRB4), Gulf Coast (MRB5), and Pacific Northwest (MRB7) Major River Basins (26,649 in total) With Point-Source Nutrient Loads for 1992, 1997, or 2002 Calculated From U.S. Environmental Protection Agency Permit Compliance System Data (USEPA, 2006c), and the Pilot Study Facilities in the Southeast (MRB2).

The distribution of facilities for which point-source nutrient loads could be calculated varied by region due to jurisdictional differences in reporting to PCS, geographic differences in population, and differences in the types of facilities (Figure 6). The Upper Mississippi (MRB3) had the largest density of facilities, due partly to greater population densities, states with good PCS database records, and additional facility information gathered directly from states in support of SPARROW model development (Robertson and Saad, this issue). Lower densities of facilities are apparent in more western regions, where population densities are smaller and large industrial facilities are more widely distributed, but it is noteworthy that the Pacific Northwest (MRB7) had the lowest percentage of minor facilities with data in PCS. Other causes for sparse facility data can be related to management practices of facilities, especially sewage systems that do not release effluent to surface-water bodies but hold the water in ponds or redistribute the effluent to nearby uses such as irrigation of public golf courses or agriculture lands. These types of facilities were most common in the dry midwestern states of Nebraska and Kansas.

A comparison of point-source nutrient loads for a subset of facilities was made to determine if the TPC nutrient concentration data produced point-source nutrient loads that are significantly different from those calculated using facility-specific nutrient concentrations. Annual (2002) point-source nutrient loads were calculated for facilities with 12 months of effluent flow and facility-specific TN and TP concentration data, and compared to recalculated nutrient loads using TPC values for the same facilities. There are 122 facilities with sufficient monthly effluent flow and facility-specific concentration data to calculate the two sets of annual TN point-source loads, and 1,823 facilities to calculate the two sets of TP point-source loads. Most of the data in the subset were for sewage systems (107 of the 122 with TN data, and 1,418 of the 1,823 with TP data). As in McMahon *et al.* (2007), a Wilcoxon sign-rank test was used to test the hypothesis that the median difference at the 0.05 alpha level between annual TN and TP point-source loads for the two datasets was zero. Test results showed that TN point-source loads were not significantly different (*p*= 0.26). Similarly, statistics for the TP point-source loads from the two datasets were not significantly different (*p*= 0.59). The total annual point-source nutrient loads calculated from the facility-specific effluent flow and TN or TP concentrations data were larger than the total annual point-source nutrient loads calculated using the TPC concentrations. Similarly, McMahon *et al.* (2007) concluded that in MRB2, the TP point-source loads from TPC concentrations were more conservative, or smaller, than point-source loads based on facility-specific data.

## Discussion

The relationship between point-source discharges and surface-water quality is well established, and knowledge of those discharges is critical to the effective management of water quality to support a variety of water uses. Point-source nutrient loads have been statistically related to local stream-water quality and to stream loads affecting downstream water bodies. Thus, there is scientific evidence that point sources and surface-water quality are linked over broad spatial scales. Generally, point-source discharges are a component of any TMDL assessment, which is the mechanism provided by the Clean Water Act for managing and restoring surface-water quality. Thus, given that point-source discharges are known to affect water quality and are typically included in water-quality management plans, it is critical to have accurate information documenting their location, and the flows and loads.

Commonly, the environmental factors leading to degraded water quality extend over large areas and multiple jurisdictions, and to develop effective management plans information describing those factors needs to be collected consistently across entire watersheds. For example, the Chesapeake Bay watershed extends over parts of six states and the District of Columbia, and restoration of water quality in the Bay requires the coordinated efforts of all of the jurisdictions as well as consistent information describing nutrient sources within their areas. Although regional databases may meet the needs of specific water bodies such as the Chesapeake Bay, the Clean Water Act is national in scope, and ideally data describing environmental factors affecting water quality would be collected consistently and made available nationally. The PCS, at the time of this study, was the only national database for storing information describing point sources and their discharges, but it was not designed with the objective of providing consistent and widely available data describing point-source nutrient loads to surface waters across the country. National regulatory databases do offer the potential for providing consistent widely available point-source data, but that objective would require modifications in the way the database is developed and maintained. Recent efforts by USEPA to develop data quality-checking and point-source load computations using data from PCS and ICIS-NPDES are definite improvements to enable the general public and resource managers to be able to use those databases effectively to compute loads, but lacking the building blocks to compute the nutrient loads still prevail if the data are not collected systematically and reported accurately across all jurisdictional boundaries.

In the course of this study, we found inconsistent listing of facilities as well as inconsistent and incomplete reporting of facility locations, effluent flows, and effluent nutrient concentrations to be limitations in the utility of the PCS database. Inconsistent listing of facilities creates significant uncertainty in evaluating point-source nutrient loads on a regional or national basis because it precludes the extrapolation of inputs based on data from other facilities to develop a full accounting for point-source nutrient loads. Lack of effluent flow further limits the utility of the data provided for many of the facilities that are listed in PCS. Commonly, detailed records of facilities and their effluent are compiled by states but are not included in PCS due to lack of resources, or to lack of procedures or regulatory requirements for doing so. The work to adapt the PCS database to meet the needs of SPARROW models might be found helpful to a broader audience and could provide alternative load estimate mechanisms to enhance USEPA's pollutant loading tool. The current lack of consistent and complete TN and TP concentrations in USEPA databases due to permitting requirements, or the lack thereof, are overcome by using TPC concentrations. Additional coordination to enhance TPC concentrations, or improve reporting of TN concentrations for all facilities would benefit both USGS and USEPA.

In the PCS database, municipal and industrial wastewater discharge facilities are designated as either major or minor facilities, based on a combination of factors that include the volume of effluent flow among other things. Major facilities typically discharge on average more than 1 mgd, and minor facilities typically discharge <1 mgd of effluent. Appropriately, information on major facilities is reported to a much greater degree than is information for minor facilities, but information for minor facilities may still be important for assessing and managing local and regional-scale water-quality issues (i.e., TMDL and nutrient criteria). Of the major facilities reported in PCS (7,174), most (74%) included effluent flow data, thus allowing the estimation of point-source nutrient loads. This is an important benefit given that the major facilities contribute a large part of the point-source nutrient loads on a regional basis. Minor facilities, however, are more numerous (111,076) but effluent flow and concentration data are lacking; however, their contribution to total nutrient loads on a regional basis can represent a significant part of the total point-source contribution of nutrient mass to surface waters of the U.S. The USGS has collected a substantial amount of additional data for facilities (both minor and major) from states and facilities, presumably not currently available on PCS or ICIS-NPDES, especially in the Upper Mississippi (MRB3). A systematic and hierarchical approach to address missing data for facilities, especially minor facilities, is being addressed by USGS to compute point-source nutrient loads for more facilities for future SPARROW models. Coordination and communication channels between USEPA and USGS are warranted here as well to share the benefits of this work.

## Summary and Conclusions

Data describing point-source discharge locations, effluent flows, and concentrations were compiled from the USEPA PCS database system for six large regions of the continental U.S., and used to calculate point-source nutrient loads to surface waters. Methods used to calculate the point-source nutrient loads were developed in a previous pilot study for the Southeastern U.S., which provided point-source nutrient loads to support the development of a TN SPARROW model for that region. The results and experiences working with the source data in this effort were similar to those in the pilot study. Discharge information in PCS is inconsistent across regions and states, and therefore required extensive corrections to the data, or required contact with state agencies and facilities to complete and correct some data entries and to gain information on coding and reporting practices, as well as on local effluent discharge practices.

Point-source nutrient loads require effluent flow and nutrient concentration data and loads were calculated only for those facilities that are listed in PCS with effluent flow, because measured flow is considered critical to accurate point-source nutrient loads. However, to maximize the number of facilities for which point-source nutrient loads could be calculated, both measured and estimated concentration values were used. Many states do not require facilities to report TN or TP effluent concentrations, and the majority of available nutrient concentration data were for TP in the discharge of major facilities, those that discharge on average more than 1 mgd of effluent. Minor facilities are those that discharge on average <1 mgd of effluent. Surrogate TPC data were used to calculate point-source nutrient loads when facility-specific nutrient effluent concentration data were missing. Concentration data from facilities considered in this study were analyzed and developed into combined regional concentrations using the population of concentrations for all facilities in the five MRBs, based on facility type, size, and season. The resulting combined MRB concentration data were compared with a national dataset of concentrations from the USEPA, which helped to produce a more robust set of TPC concentrations based on a much larger population of facility effluent concentration data. Overall, the paucity of TN concentration data was evident and required a more extensive use of surrogate TPC concentrations than was necessary for TP concentrations.

Data from about 118,250 municipal and industrial facilities (both active and inactive) were assembled for 45 states and the District of Columbia. Systematic data verification and correction efforts were performed on the data to correct erroneous facility location, and effluent flow and concentration data, as well as to fill in missing data through contacts with state agencies and facility operators. From the total population of facilities (118,250), annual point-source nutrient loads for over 26,600 facilities were calculated for at least one of the three years of interest (1992, 1997, and 2002). Annual point-source nutrient loads for 2002 were calculated for almost 66% of major and 14% of minor facilities. Regions in the U.S. that are densely populated or have large concentrations of industrial and municipal facilities, such as the New England and Mid-Atlantic (MRB1) and the Upper Mississippi (MRB3), produced the largest point-source nutrient loads on both a total mass and on a mass per area basis. Sewage systems contributed the greatest share of point-source nutrient loads overall, and their percent contribution of total annual point-source nutrient loads by region ranged from 27% for TP in the Missouri (MRB4) to 86% for TN in the New England and Mid-Atlantic (MRB1).

Overall, there are many more minor than major facilities, but reporting of discharge information for the major facilities is more complete. On a regional basis, major facilities contributed the greatest mass of nutrients to surface waters, but minor facilities contributed an appreciable percentage of the mass of nutrients, ranging from as little as 2% in the New England and Mid-Atlantic (MRB1) up to almost 60% in the Missouri (MRB4) of the total TP, and from almost 6% in the Pacific Northwest (MRB7) up to almost 50% in the Missouri (MRB4) of the total TN. Thus, although minor facilities have few data in PCS that is necessary to compute point-source nutrient loads, our work indicates fuller documentation of point-source nutrient loads from minor facilities is warranted in regional water-quality assessments because of the apparent aggregate contributions of nutrients from minor facilities in some regions.

The work described in this paper represents an initial step toward future efforts to develop a complete and consistent database documenting point-source nutrient loads for individual facilities in the U.S. Such a database is needed to provide a nationally consistent basis from which to evaluate the role of point-source discharges on surface-water quality at both national and regional scales.

## SUPPORTING INFORMATION

**Table S1.** Standard Industrial Classification (SIC) codes (Office of Management and Budget, 1987).

Please note: Neither AWRA nor Wiley-Blackwell is responsible for the content or functionality of any supporting materials supplied by the authors. Any queries (other than missing material) should be directed to the corresponding author for the article.

## References

[b1] GAO (General Accounting Office) (2000). Water Quality: Key EPA and State Decisions Limited by Inconsistent and Incomplete Data.

[b2] Gianessi LP, Peskin HM (1984). An Overview of the RFF Environmental Data Inventory: Methods, Sources and Preliminary Results.

[b3] Hoos AB, Terziotti S, McMahon G, Savvas K, Tighe KC, Alkons-Wolinsky R (2008). Data to Support Statistical Modeling of Instream Nutrient Load Based on Watershed Attributes, Southeastern United States, 2002.

[b4] Hummel PR, Kittle JL, Duda PB, Patwardhan A (2003). Calibration of a Watershed Model for Metropolitan Atlanta.

[b5] Luken RA, Basta DJ, Pechan EH (1976). The National Residuals Discharge Inventory: An Analysis of the Generation, Discharge, Cost of Control, and Regional Distribution of Liquid Wastes to be Expected in Achieving the Requirements of Public Law 92-500.

[b6] McMahon G, Tervelt L, Donehoo W (2007). Methods for Estimating Annual Wastewater Nutrient Loads in the Southeastern United States.

[b7] Moore RB, Johnston CM, Robinson KW, Deacon JR (2004). Estimation of Total Nitrogen and Phosphorus in New England Streams Using Spatially Referenced Regression Models.

[b8] NOAA (National Oceanic and Atmospheric Administration) (1999). Coastal Assessment and Data Synthesis (CA&DS) Framework, National Coastal Assessment (NCA) Branch, Special Projects (SP) Office, National Ocean Services (NOS).

[b9] Office of Management and Budget (1987). Standard Industrial Classification Manual.

[b10] Preston SD, Alexander RB, Schwarz GE, Crawford CG Factors Affecting Stream Nutrient Loads: A Synthesis of Regional SPARROW Model Results for the Continental United States. Journal of the American Water Resources Association.

[b11] Preston SD, Alexander RB, Woodside MD, Hamilton PA (2009). SPARROW MODELING – Enhancing Understanding of the Nation's Water Quality.

[b12] Preston SD, Brakebill JW (1999). Application of Spatially Referenced Regression Modeling for the Evaluation of Total Nitrogen Loading in the Chesapeake Bay Watershed.

[b13] Robertson DM, Saad DA Nutrient Inputs to the Laurentian Great Lakes by Source and Watershed Estimated Using SPARROW Watershed Models. Journal of the American Water Resources Association.

[b14] Smith RA, Schwarz GE, Alexander RB (1997). Regional Interpretation of Water-Quality Monitoring Data. Water Resources Research.

[b15] USC (United States Code) (2002). Federal Water Pollution Control Act (a.k.a. Clean Water Act).

[b16] USDA (U.S. Department of Agriculture) (1994). 1992 Census of Agriculture, United States Summary and State Data.

[b17] USDA (U.S. Department of Agriculture) (1998). 1997 Census of Agriculture, United States State Level Data.

[b18] USDA (U.S. Department of Agriculture) (2004). 2002 Census of Agriculture, United States Summary and State Data.

[b19] USEPA (U.S. Environmental Protection Agency) (1988). Summary of Effluent Characteristics and Guidelines for Selected Industrial Point Source Categories: Industry Status Sheets, Washington, DC.

[b20] USEPA (U.S. Environmental Protection Agency) (2000). Nutrient Criteria Technical Guidance Manual – Rivers and Streams.

[b21] USEPA (U.S. Environmental Protection Agency) (2006a). Reassessment of Point Source Nutrient Mass Loadings to the Mississippi River Basin, November, 2006, Mississippi River/Gulf of Mexico Watershed Nutrient Task Force.

[b22] USEPA (U.S. Environmental Protection Agency) (2006b). Gulf Hypoxia Action Plan 2008.

[b23] USEPA (U.S. Environmental Protection Agency) (2006c). Envirofacts Data Warehouse, Permit Compliance System.

[b24] USEPA (U.S. Environmental Protection Agency) (2007). National Water Quality Inventory: Report to Congress, 2002 Reporting Cycle.

[b25] USEPA (U.S. Environmental Protection Agency) (2009). An Urgent Call to Action – Report of the State-EPA Nutrient Innovations Task Group.

[b26] Zogorski JS, Blanchard SF, Romack RD, Fitzpatrick FA (1990). Availability and Suitability of Municipal Wastewater Information for Use in a National Water-Quality Assessment: A Case Study of the Upper Illinois River Basin in Illinois, Indiana and Wisconsin.

