# Supplementary material for: Nutrient Loadings to Streams of the Continental United States from Municipal and Industrial Effluent
Source: J Am Water Resour Assoc. 2011 Oct;47(5):950–64. doi: 10.1111/j.1752-1688.2011.00576.x (PMC3307619; doi:10.1111/j.1752-1688.2011.00576.x)
Supplement: Supplementary file 1 [file jawr0047-0950-SD1.pdf]

Table S1. Standard Industrial Classification (SIC) codes (Office of Management and Budget, 1987).

SIC code	SIC description
0211	Beef Cattle Feedlots
0213	Hogs
0251	Broilers, Fryers, and Roaster Chickens
0252	Chicken Eggs
0253	Turkey and Turkey Eggs
0254	Poultry Hatcheries
0259	Poultry and Eggs, NEC
0279	Animal Specialities, NEC
0921	Fish Hatcheries and Preserves
1221	Bituminous Coal and Lignite Surface Mining
1311	Crude Petroleum and Natural Gas
1389	Oil and Gas Field Services, NEC
1475	Phosphate Rock
1541	General Contractors-Industrial Buildings and Warehouses
2011	Meat Packing Plants
2015	Poultry Slaughtering and Processing
2046	Wet Corn Milling
2082	Malt Beverages
2085	Distilled and Blended Liquors
2611	Pulp Mills
2621	Paper Mills
2631	Paperboard Mills
2812	Alkalies and Chlorine
2819	Industrial Inorganic Chemicals, NEC
2821	Plastics Material and Synthetic Resins, and Nonvulcanizable Elastomers
2823	Cellulosic Manmade Fibers
2824	Manmade Organic Fibers, Except Cellulosic
2851	Paints, Varnishes, Lacquers, Enamels, and Allied Products
2864	Cyclic Organic Crudes and Intermediates, and Organic Dyes and Pigments
2869	Industrial Organic Chemicals, NEC
2873	Nitrogenous Fertilizers
2874	Phosphatic Fertilizers
2899	Chemicals and Chemical Preparations, NEC
2911	Petroleum Refining
3312	Steel Works, Blast Furnaces (Including Coke Ovens), and Rolling Mills
3334	Primary Production of Aluminum
3339	Primary Smelting and Refining of Nonferrous Metals, Except Copper and Aluminum
3443	Fabricated Structural Metal Products (Boiler Shops)
3589	Service Industry Machinery, NEC
4941	Water Supply
4952	Sewage Systems
4953	Refuse Systems
4959	Sanitary Services, NEC
5171	Petroleum Bulk Stations and Terminals
6513	Operators of Apartment Buildings
6515	Operators of Residential Mobile Home Sites
6552	Land Subdividers and Developers, Except Cemeteries
7011	Hotels and Motels
7032	Sporting and Recreational Camps
7033	Recreational Vehicle Parks and Campsites
7999	Amusement and Recreation Services, NEC
8062	General Medical and Surgical Hospitals

8211	Elementary and Secondary Schools
8221	Colleges, Universities, and Professional Schools
8222	Junior Colleges and Technical Institutes
8231	Libraries
8249	Vocational Schools, NEC
8351	Child Day Care Services
8361	Residential Care
8422	Arboreta and Botanical or Zoological Gardens
9223	Correctional Institutions
9621	Regulation and Administration of Transportation Programs
9711	National Security
